# The Origin of Medical Science

**Published:** 1872-05-15

**Authors:** 


### THE ORIGIN OF MEDICAL SCIENCE.

The following interesting address on "The Origin of Medical Science," was delivered by Dr. B. F. Dawson, of the *American Journal of Obstetrics*, before the Association of American Medical Editors:

Members of this Association: we meet this evening to fraternize in the most emphatic acceptation of the word — to celebrate the third anniversary of our association; to renew old friendships, and to behold once more, however changed, the faces of those we delight to see, and in whose eyes beams the light of a hearty recognition and welcome.

In accordance with the example set by my distinguished predecessor, as President of this Association, it devolves upon me to entertain you, if possible, by an annual address.

Much as I feel, and fully as I appreciate the honors of the office to which I was elected last year, yet I also feel that the position I at this present moment occupy is not among those professional distinctions either covetously sought after, or earnestly desired.

On the contrary, among the members of our profession, at least, I am quite sure such a task is more frequently shunned than desired. For myself, at least, I plead no exemption from this possible frailty.

Leaving topics of direct interest to this Association for our business meetings, I propose to engage your attention for a short time on a subject of interest to you as scholars and disciples of Æsculapius, to wit, "The Origin of Medical Science."

The obscurity which surrounds the early infancy of the art of medicine, envelopes also the origin of all branches of human knowledge. All we do know is that from the earliest periods of history it was practiced; and from this fact we may believe that at the dawn of the arts it had obtained a place among them.

Reasoning from the general course of nature, it is evident that ancient man was subject to various influences affecting his physical health, and that therefore he must very soon have been obliged to seek for means of alleviating the pains, and curing the disorders with which he was afflicted. From this reasoning, therefore, we may safely affirm that the treatment of disease bears almost as ancient a date as the origin of man himself.

Among many rude and savage races of the present day, as those of Africa, Australia, North America, and Greenland, traces are found of the practice of medicine and surgery. We are told that those savages know how to distinguish different diseases, and to apply a more or less appropriate remedy. These uncivilized tribes, therefore, may be taken by us as the picture of mankind in their original primitive condition.

Allowing that ancient man had diseases which he naturally sought to cure or alleviate, yet we must presume that the means and methods were crude, and more frequently the result of fortunate accidents than of rational investigation and study. Receiving successively by tradition a knowledge of such discoveries, it is probable that each tribe made new additions to such knowledge, and in this manner their acquisitions would gradually increase. The art of healing may, therefore, be considered to have thus had its origin.

Later on in the history of man, when tribes became divided into communities, and communities into distinct families, it is probable that medicine took a more established form. Each family had its own traditions and particular mode of alleviating disease; and thus successively they profited by the experience that had been collected by their predecessors. Still later, as the different arts developed their infant life, men of power and influence, who were desirous of adding dignity to wealth, and to become useful to their fellow-men, naturally cultivated each with ardor, and were far from neglecting that of medicine, which afforded them the means of frequently rendering very necessary assistance.

Among the distinguished Grecians especially, the art of healing was largely studied, and the names of Chiron, Aristacus, Theseus, Telamon, Patrocles, Ulysses, and some others, we find were no less honored in Greece for their medical knowledge than for those exploits which, whether fictitious or real, have conferred a durable celebrity to their names.

Among the writings of the ancient poets, we also find conclusive proofs that they likewise applied themselves to the study of medicine, for we find them sometimes embodying in their works whatever they considered from experience to be valuable medical precepts. In such early periods, when the art of writing was but little diffused, or perhaps almost unknown, the measures and melodious rhythm of poetry were extremely valuable for impressing the mass with certain truths. Orpheus and others have sung of "*the beneficent art which prolongs life, allays pain, and by restoring health gives happiness and pleasure.*"

In the writings of Homer we find him frequently speaking of the wounds of his heroes and giving advice as to the dressing of wounds and the action of several remedial agents. He tells us that wine was applied to the wounds of the soldiers composing the army of the Greeks before Troy, and that the knife of the surgeon was frequently resorted to. From these facts, therefore, we are entitled to infer that these practices are of a date prior to the age in which he lived.

Next to the poets, we find the ancient priests combining the art of healing with their other instruments of power. Indeed, the medical and sacerdotal professions have in reality many points of resemblance. Both bring into action the same principles — hope and fear; and although the objects of these two passions are not the same in the hands of the priest as in those of the physician, their effects had, at that time, nearly the same degree of influence in promoting the doctrines of both. Certain it is that medicine, like superstition, exerts upon the minds of men an influence proportional to their weakness. In short, no art penetrates further into the human heart; no profession enables its votaries more easily to obtain possession of the most important family secrets, and no species of doctrine has a stronger voice of appeal to the public credulity. It was not, therefore, surprising to find the early priesthood practicing the healing art. From this time forward, we find medicine and religion traveling hand in hand. The ancient gods were worshiped for the cures effected in their name by the priests, and which were proclaimed by them to depend upon their intercourse with the Deity.

The Jewish priests appear to have been the only physicians of their nation. It was to them, we learn, that the people addressed themselves for the cure of leprosy; and it was left them to decide upon the fate of the families and individuals who were attacked with disease. In the porch of the Temple of Jerusalem, we are told that a complete formulary of remedies was exhibited.

It was among the ancient Egyptians, however, that the priests carried this power to the greatest perfection. They alone became the teachers, as well as the practitioners of medicine — in fact, they constituted an exclusive learned sect. The art of medicine was taught in their temples with those mysteries of initiation which are calculated to create believers. They subjected the art to absurd regulations, as, for instance, fixing a law to determine the time for the application of remedies in *all diseases, without any discrimination;* dividing the art into as many different branches as there were diseases, or organs in the body, and finally, compelling the son to follow the profession of his father.

In ancient Greece, also, medicine was cultivated in the temples ; and many of their deities were supposed to possess the power of healing the sick. Apollo, Diana, Minerva, and Juno, besides their prophetic powers, through their priests, had assigned to them the exercise of particular branches of the art.

About this time Æsculapius acquired the ascendancy over all other deities as a god-physician, and was called the son of Apollo by the priests. Aristophanes informs us of the manner in which this deity performed his cures. Those who came to consult him were first obliged to purify themselves with water. They then deposited their offerings upon his altar, and reposed upon beds placed in the middle of the temple. As soon as they fell asleep, a priest clothed in the dress of Æsculapius, and accompanied by his daughters, entered and informed each of his remedy. As the god was not to be revealed but in a dream, the patients crouched themselves upon the skins of the sacrificed rams, in order that they might secure celestial visions. Not to feign the most profound sleep, even when awake, was an unpardonable crime. Nor was it considered less dangerous to doubt that what they either saw or heard was by heavenly inspiration. If the advice thus given did not always restore health, it was at least becoming that the patients should not contract any new disorder. In consequence of some prudent precautions in this respect, many cures must have been accomplished by the diversion which the patients enjoyed in the course of their journeys to the temples, and the not less invigorating effects of hope.

Of the numerous temples dedicated to Æsculapius, the most celebrated were those of Epidaurus, Pergamos, Cos and Cnidos. The walls and pillars of the latter temple were covered with inscriptions describing the history of diseases, and giving an account of the remedies, which had been successfully used in their cure, as advised by the deity. We are told that many people had these engraved upon metal, stone, or wood, for ready reference. However imperfect and unreliable these must have been, they must nevertheless have made a valuable collection for that period. They formed, indeed, as it were, the first rudiments of the art of healing, and were the first steps in the direction of that method of observation and research which has steadily augmented up to its present vast proportions, and has placed the science of medicine on a solid basis.

In course of time those temples, situated at Cos and Cnidos, were considered schools for the study of medicine. Both of them flourished together for a considerable period, and between them ultimately sprang great rivalry. To this jealousy may be ascribed the sudden impetus given to the medical art at that period.

Hitherto, as we have seen, the physicians of the several periods were heroes, poets and priests—more deserving the name of empirics. They observed diseases and symptoms, and tried various remedies, and more or less judged from analogy. Their theory, which was as vague as their practice, was undetermined. The ignorance of the people had made it easy to satisfy them with any form of the art, and the credulity of the same had accustomed the more enlightened class to practice deception under the guise of learning.

But in time certain men of more exalted character began to direct their attention to the study of the arts, and, among others, to that of medicine. The revolution which these early philosophers effected in the healing art was a work of necessity. The time had arrived when it was no longer to be kept locked in the mysteries of the temples, and for placing it in the possession of others than the priesthood. From that time forward, therefore, a rational system of medicine was rapidly substituted for the existing unreliable and primitive rules, and the healing art was clothed with the same importance and dignity that graced the other branches of human knowledge.

These philosophers then freed the art of medicine from the superstitious and hypocritical character it had hitherto possessed. They transformed an occult and sacerdotal doctrine into a popular science and a common art.

Although these "reformers" did this much, yet they themselves committed many errors. Not satisfied with elevating medicine to the equality of other arts, they attempted also to transfer to it the imaginary laws of their system of natural philosophy, which was fertile with errors and conjectures.

Thus, Pythagoras as an example, we learn endeavored to explain the laws of animal economy, the causes of disease, and the action of medicines by the power of numbers; Democritus again referred them to the motion and position of the atoms of matter; while Heraclitus attempted to account for them by the influence of the fire of the universe. From such teachings naturally arose many of the absurd theories of which we find examples in the works of Plato, Aristotle and

Plutarch, and to a slight extent in the writings of Hippocrates also.

Of all the philosophers who at that time devoted themselves to the study of medicine, Acron of Agrigentum, in Sicily, is said to have taken the lead as the boldest and most original. Being convinced that the art of medicine should be based upon experience only, he accordingly endeavored to teach that, by studying and appreciating the different symptoms of disease by analogy, it was not difficult to find out the indications of cure. Wise as Acron was considered in his day, and enjoying as he did a distinguished reputation, yet he found it impossible to wholly overcome certain positive and dogmatical theories, and it was only long after his death that his views were accepted by a certain sect of physicians.

We have seen, then, that the philosophers of antiquity both improved and injured the science of medicine. Rescuing it from undiscerning ignorance, they, however, threw into it almost as fallacious conjectures; they delivered it over from the blindness of empiricism to all the rashness of dogmatism.

In the hands of the ancient poets we have seen that the art of healing was simply poetical sentiments; in those of the ancient priests we found it clothed with the vague and mysterious power of superstition; and lastly, in the hands of the primitive philosophers, though its scattered and undigested materials were combined and formed into more or less regular and perfect systems, yet it still was overrun with the weeds of many crude and illogical reasonings, and could hardly claim a just right to be considered a science. Such was the condition of medicine at the period rendered famous by the appearance of Hippocrates. His ancestors, we are told, during seventeen generations, in regular succession, from father to son, had followed the profession of medicine in the island of Cos. Surrounded from infancy with all the objects of his studies; endowed by Nature with a genius which was both comprehensive, penetrating, bold and prudent, he commenced his career under the most favorable auspices, and pursued it during more than eighty years, with that degree of renown which was equally due to his talents and his character.

The date of the appearance of the "Father of Medicine," as he is called, on this terrestrial globe, is fixed at 460 years before the advent of our Savior. Since that momentous year for the science of medicine, more than twenty-three centuries have rolled from time into eternity, and yet still the name of Hippocrates is not the less known or honored. What made him so great is well known to all of you, and what he did for the science of medicine is familiar to each one of us. I will not, therefore, weary you with its recitation. On the contrary, it is my purpose to leave those 2,300 years of time, with all their glory, undisturbed, and ask you rather to look upon the science of medicine as it exists at present, and in the picture thus presented behold the mighty edifice of our life's profession, as it stands to-day, reflecting from its resplendent walls in golden beams the truths of a science that has inspired some of the noblest hearts that ever beat to worship at her shrine, and bears inscribed upon her tablets names of some of the world's truly great and good men.

Ancient as the science of medicine has been shown to be, yet how little does this colossal temple of to-day resemble the rough and unhewn stones of its infancy. The weak and uncertain steps with which it at first essayed to walk, have given place to those stupendous strides which even in our short life we have seen it take. Rising from the chains and trammels of ignorance and superstition, we behold it to-day standing erect and firm by its sister sciences, "clothed in purple and fine linen."

Looking upon the science of medicine of to-day, and recalling some of those important advances which it has made within comparatively a few years, it is not difficult to prophesy as to the future. The physicians and surgeons of distinction whose names are honored among us, are but the predecessors of equally brilliant men who shall strive, as these have striven, in future years, to develop new truths from the inexhaustible treasures of research and study.

Inexhaustible, indeed, is the well of medical science. Each new year brings us new volumes of medical lore, and gives birth to some new and brilliant genius among the disciples of Æsculapius, and yet the cry is "still they come."

And what is the future of medical science? Look back upon the imperfect sketch that I have just given you of the weakness of its infant life; see it now before you in all the grand vigor of its manhood, and your cheeks wlll burn with the blush of pride, and the convictions of your own hearts reply that its future will be superbly grand. Let us then, my fellow editors, feel a just pride in our calling, and by that all powerful voice, "The Press," instruct our fellow worshipers at the shrine of Æsculapius, by spreading the truths of medical science over the land, and whispering in their ears those stirring and beautiful lines of Longfellow:

"Lives of great men all remind us
We can make our lives sublime,
And, departing, leave behind us
Footprints on the sands of time.

"Let us, then, be up and doing,
With a heart for any fate;
Still achieving, still pursuing,
Learn to labor and to wait."

